# Evaluating Host Defense Peptides: A Comparative Analysis of Synthetic Peptides and Recombinant Concatemers

**DOI:** 10.3390/biom15070980

**Published:** 2025-07-08

**Authors:** Cristina Saubi, José Vicente Carratalá, Roberto Bello-Madruga, Adrià López-Cano, Susanna Navarro, Anna Arís, Elena Garcia-Fruitós

**Affiliations:** 1 IRTA, Ruminant Production, Torre Marimon, 08140 Caldes de Montbui, Catalonia, Spain; cristina.saubi@irta.cat (C.S.); jose.carratala@irta.cat (J.V.C.); adrialopezcano@gmail.com (A.L.-C.); 2 The Systems Biology of Infection Laboratory, Department of Biochemistry and Molecular Biology, Biosciences Faculty, Universitat Autònoma de Barcelona, 08193 Cerdanyola del Vallès, Spain; roberto.bello@uab.cat (R.B.-M.);

**Keywords:** host defense peptides, recombinant production, concatemers, antimicrobial activity, *Lactococcus lactis*

## Abstract

The global antibiotic resistance crisis raises concerns about antibiotic use, and alternative strategies are urgently needed. In this context, host defense peptides (HDPs) have rapidly gained interest. However, one of the main obstacles is their production strategy. Chemical synthesis is the most widely used, although it is not scalable and has sequence limitations. A possible alternative is recombinant production, but the strategies used so far have limited efficiency. In this study, we aim to compare the activity and main characteristics of different HDPs produced by both chemical synthesis and by recombinant production, using an approach based on tetramers to ameliorate the production process. The results obtained showed that the production of HDPs as tetrameric peptides by recombinant production in *Lactococcus lactis* enhanced the peptide activity, with HDPs being much more active in terms of antimicrobial activity, more structurally stable, and nanostructured. Thus, the recombinant strategy described herein, fusing four repetitions of the same peptide, can become a real alternative to produce highly active HDPs through a scalable production process.

## 1. Introduction

Bacterial infections pose a significant global health burden and are the primary cause of infection-related deaths globally, with wide-ranging effects on human, animal, and environmental health [1,2,3,4]. Their consequences are exacerbated by the rapid emergence of multidrug-resistant bacteria, which leads to severe strain on healthcare systems and worsens economic instability and social disparities [3,5,6,7].

Over the past two decades, host defense peptides (HDPs) have rapidly gained significant interest as alternatives to traditional antibiotics [8,9,10,11]. These peptides are characterized by their short length, typically less than 50 residues, are amphipathic and cationic under physiological conditions [11,12]. HDPs have demonstrated a wide range of antimicrobial and antibiofilm activities, including direct pathogen-killing through membrane disruption and inhibition of metabolic pathways [12,13,14,15]. Additionally, HDPs can neutralize bacterial endotoxins, such as Gram-negative lipopolysaccharides (LPS) [16,17], and exhibit immunomodulatory activities in vertebrates [18,19,20]. The ubiquitous presence of HDPs across all animals and plants as components of defense mechanisms, coupled with their multifunctional nature, makes them a promising solution to the antibiotic resistance crisis [11].

One of the primary obstacles in advancing HDP-based therapies is developing production strategies that ensure the proper peptide conformation and activity while being cost-effective, scalable, and complying with safety standards if intended for therapeutic use.

Chemical synthesis, particularly solid-phase peptide synthesis (SPPS), is the most common strategy for peptide production. In SPPS, the peptide is anchored by its C-terminal to a resin support, allowing for the automated sequential addition of amino acids [21,22]. The complete peptide is then released from the solid support, producing a homogeneous product with high purity [22,23,24]. However, additional synthetic steps are required to add post-translational modifications (PTMs), such as disulfide bonds, glycosylations, and methylations, increasing both cost and complexity [21,23,24,25,26].

Major disadvantages of SPPS are elevated costs, inefficient scalability, environmental concerns due to toxic reagents, and sequence limitations. The efficiency of each coupling step declines with each cycle, resulting in lower yields and more side products in long peptides [23,24]. Moreover, certain sequences can be difficult to synthesize and require further optimization, for example, those rich in amino acids with reactive side chains such as cysteines, histidines, or aspartic acid [25].

A cost-effective alternative is recombinant production in heterologous systems [27]. It is based on DNA technology to modify cells, turning them into factories for synthesizing exogenous peptides or proteins, which are purified in subsequent steps. This method is widely used in research and industry, and is scalable [27,28]. However, peptides have reduced stability and are more prone to protease degradation than proteins, impairing their recombinant production [29,30,31]. An added challenge, specific to HDPs, is their potential toxicity to the cell factory, directly impacting the production yield [32,33]. Because of these, recombinant strategies frequently require expressing HDPs with fusion tags to improve their stability, facilitate purification, and reduce toxicity toward the host bacterial cells [33,34]. These tags are then often cleaved to avoid masking the biological activity of HDPs [35,36].

Post-translational modifications of recombinant peptides vary depending on the cell factory used. Mammalian cells can perform more intricate PTMs than bacteria, but the process is more costly and complex [33]. *Escherichia coli*, a Gram-negative bacterium, is the most commonly used and optimized cell factory due to its simplicity and efficiency [33,37,38]. Despite its simpler PTM capabilities, various HDPs have been successfully produced in *E.* coli using different design strategies: cathelicidins (e.g., human LL-37 [39], goat ChMAP-28 and proline-rich mini-ChBac7.5Nα, [40], murine CRAMP [41], and bovine BMAP28 [42]) and several defensins (such as human α-defensin 5 [43], human β-defensin 6 [44], bovine neutrophil β-defensin 12 [45], and bovine lingual antimicrobial peptide [43], among others).

Recombinant production often results in lower purity levels compared to chemical synthesis due to the presence of by-products from the cell factory in the final product [21]. Specifically, in the case of *E. coli*, endotoxins such as LPS can pose significant safety risks, necessitating additional purification steps to ensure a safer product [34,46]. However, these extra steps can reduce the overall yield of the process. An attractive approach could be the use of generally regarded as safe (GRAS) organisms, such as the Gram-positive *Lactococcus lactis*, which are free from endotoxins [47,48]. There are only a few examples in the literature of HDPs produced in *L. lactis*: human β-defensin 1 [32], human LL37 [49], bovine BMAP27 [50], and a multidomain protein of avian β-defensins [51].

Despite these considerations, there is limited research directly comparing the effects of these different production systems on the final product’s properties. Harder et al. concluded that the antimicrobial activity and biochemical properties of human β-defensin 3 were preserved when produced either synthetically or recombinantly in *E. coli* [52]. Similar results were observed when studying a short cysteine-rich insecticidal spider peptide [35] and indolicidin [53]. However, further efforts were required to ensure the formation of disulfide bonds and native folding of the peptide, independently of the production method.

In this paper, we aim to compare the effects of using two different production strategies—chemical SPPS and recombinant production in *L. lactis*—of four bovine HDPs: bovine neutrophil β-defensin 1 (BNBD1), bovine neutrophil β-defensin 3 (BNBD3), bactenecin 5 (Bac5), and the bovine myeloid antibacterial peptide 27 (BMAP27). To improve the efficiency of recombinant production and avoid the cleavage of fusion tags, the recombinant version of the peptides was produced as tetrameric multidomain fusion proteins. The synthetic peptides were compared to their recombinant counterparts in terms of biological activity (antimicrobial, antibiofilm, and endotoxin-binding) and structure (oligomerization state and folding).

## 2. Materials and Methods

### 2.1. Bacterial Strains

*Lactococcus lactis* NZ9000 (*pepN::nisRnisK*, MoBiTech, Hamburg, Germany) was used for recombinant protein expression. The antimicrobial activity was evaluated on the Gram-positive methicillin-sensitive *Staphylococcus aureus* (MSSA, ATCC-35556, LGC Standards S.L.U., Barcelona, Spain) and Gram-negative *Pseudomonas aeruginosa* (CECT4122, Valencia, Spain). *L. lactis* was grown in M17 (Sigma-Aldrich, Madrid, Spain) supplemented with 0.5% glucose, MSSA in Brain Heart Infusion (BHI; Scharlau, Sentmenat, Spain), and *P. aeruginosa* in Luria-Bertani (LB).

### 2.2. Synthesis of Host Defense Peptides

Two types of molecules were produced: synthetic host defense peptides (HDPs) and multidomain recombinant proteins based on the same HDPs. For this, the mature sequences of bovine neutrophil β-defensin 1 (BNBD1, UniProt_P46159) and 3 (BNBD3, UniProt_P46161), as well as cathelicidins 2 (Bac5, UniProt_P19660) and 6 (BMAP27, UniProt_P54228) were used. The tertiary structures of monomeric and multidomain variants are shown in Supplementary Figures S1 and S2.

### 2.3. Synthetic Peptides

Peptides were synthesized by RoyoBiotech (Shanghai, China) in the linear form and named sBNBD1, sBNBD3, sBac5, and sBMAP27. Final peptides were not amidated at the C-terminus, and the cysteines of β-defensins were not protected.

### 2.4. Recombinant Production of Proteins

#### 2.4.1. Protein Design and Cloning

Recombinant proteins were designed as tetrameric repeats of a single HDP fused with the linker “GGSSRSS”. A C-terminal hexa-histidine (H6)-tag was added for protein purification purposes. The final constructs were BNBD1-BNBD1-BNBD1-BNBD1-H6 (rBNBD1x4), BNBD3-BNBD3-BNBD3-BNBD3-H6 (rBNBD3x4), Bac5-Bac5-Bac5-Bac5-H6 (rBac5x4), and BMAP27-BMAP27-BMAP27-BMAP27-H6 (rBMAP27x4). The genes were synthesized and their codon usage was optimized for expression in *L. lactis* by GeneArt (GeneArt^®^ Life Technologies, Regensburg, Germany). The four constructs were cloned into the chloramphenicol-resistant (Cm^R^) vector pNZ8148 (MoBiTech, Goettingen, Germany) as previously described [48]. For that, two restriction sites were added to flank the gene sequence: *NcoI* at the 5′ end and *XbaI* at the 3′ end. The resulting plasmid was transformed into *L. lactis* strain NZ9000 (*pepN::nisRnisK*) by electroporation. Clones were double validated by plasmid digestion with NcoI (FD0575, Thermo Scientific, Madrid, Spain) and XbaI (FD0684, Thermo Scientific, Madrid, Spain), and plasmid sequencing.

#### 2.4.2. Protein Expression and Purification

For protein production, clones were grown to the exponential phase (OD_600_ = 0.5–0.6) at 30 °C in 5 L of M17 media supplemented with 0.5% glucose and 5 μg/mL Cm. The intracellular protein expression was induced with 12.5 ng/mL nisin. Expression kinetics were studied to determine the optimal expression time for each protein: 1 h for rBNBD1x4 and rBac5x4, but 3 h for rBNBD3x4 and rBMAP27x4. Bacterial cells were centrifuged at 6200× *g* for 15 min at 4 °C, and the resulting pellet was stored at −80 °C until protein purification and was then resuspended in binding buffer (20 mM Tris, 500 mM NaCl, 20 mM imidazole, pH = 7.4). For protein extraction, cells were disrupted with a high-pressure homogenizer (Constant Systems CF1 Cell Disrupter, Daventry, United Kingdom) operating in continuous flow for two cycles at 40 kpsi. The soluble fraction was obtained by centrifuging at 15,000× *g* for 45 min at 4 °C and was filtered through a 0.22 μm filter. The samples were kept refrigerated throughout the process. Protein purification was performed using immobilized metal affinity chromatography (IMAC) HisTrap HP columns with the ÄKTA Start protein purification system (Cytiva, Hospitalet de Llobregat, Spain) as detailed in [43] but adjusted for 5 mL columns. The eluted fractions were dialyzed against 0.01% acetic acid and lyophilized (Telstar LyoQuest lyophilizer, Terrassa, Spain) at −60 °C and a gradual decrease in pressure of 1 mbar for 4 h, 0.5 mbar for 4 h, 0.3 mbar for 2 h, and 0.1 for 8 h. Lyophilized protein samples were stored at −20 °C and before use, resuspended with 20 mM HEPES (pH 5.2) in water endotoxin-free and quantified using a Qubit^TM^ protein assay kit (Q33211, Invitrogen, Inchinnan, UK). Protein purity was tested on SDS-PAGE electrophoresis (TGX^TM^ FastCas^TM^, Bio-Rad, Hercules, CA, USA) with Coomassie staining and Western Blot.

### 2.5. Antimicrobial Activity

#### 2.5.1. Antimicrobial Killing Assay

BacTiter-Glo^TM^ Microbial Cell Viability assay (Promega, Madison, WI, USA), which quantifies bacterial viability based on ATP levels, was used to test the bactericidal effect of HDPs at 4, 1, and 0.1 μM. MSSA and *P. aeruginosa* were grown on BHI or LB agar plates, respectively, at 37 °C overnight. A bacterial cell suspension was prepared at 2 × 10^8^ CFU/mL in protein buffer 20 mM HEPES (pH 5.2). Equal volumes of bacterial suspension and protein (75 μL) were mixed in sterile 96-well round-bottom polypropylene plates (Corning Costar, Madrid, Spain). Bacteria with protein buffer were used as a negative control; the peptide or protein alone with protein buffer was used as a protein control; and wells filled with buffer alone were used as a blank and sterility control. After a 4 h incubation at 37 °C, 100 μL of each sample was transferred into a white 96-well flat-bottom polypropylene plate (Thermo Fisher, Madrid, Spain). The plate was tempered for 5 min at room temperature before adding 100 μL of BacTiter-Glo reagent mix following the product instructions. The luminescence was measured with a Lumistar plate reader (LUMIstar^®^ Omega, v5.50R4. BMG Labtech, Ortenberg, Germany). Each sample was run in triplicates and bacterial survival was calculated using bacterial cells treated with protein buffer alone as a control using the following formula:

(1) $$Bacterial\;survival\left(\%\right)=\frac{\left(Sample-Blank\right)}{\left(Negative\;control-Blank\right)}\times 100$$

#### 2.5.2. Determination of the Minimal Inhibitory Concentrations

A 2-fold serial dilution of the peptides/proteins was performed in 20 mM HEPES (pH 5.2) and a bacterial suspension of 1 × 10^6^ CFU/mL (MSSA or *P. aeruginosa*) was prepared in 20% Mueller Hinton Broth II (MHB II, Sigma-Aldrich, Madrid, Spain) from bacterial colonies. In a 96-well round-bottom polypropylene plate (Corning Costar, Madrid, Spain), 50 μL of bacterial suspension, 15 μL of distilled water, and 35 μL of treatment or protein buffer (negative control) were mixed. The treatments’ final concentration ranged from 16 to 0.0625 μM, and a final bacteria concentration of 5 × 10^5^ CFU/mL in 10 mM HEPES and 10% MHB II. The same media alone was used as a sterility control and a water barrier in surrounding wells was created to prevent excessive evaporation. Plates were incubated for 24 h at 37 °C and a visual reading of the plate was performed. The minimum inhibitory concentration (MIC) was determined, being the lowest treatment concentration with no visual bacterial growth. All samples were run in duplicates.

#### 2.5.3. Antibiofilm Assay

*P. aeruginosa* was grown in LB supplemented with 2% glucose and 200 μL/well of bacterial suspension at OD_600_ = 0.01 (~1 × 10^7^ CFU/mL) was added in a 96-well flat-bottom polypropylene plate (Eppendorf, Madrid, Spain) with 200 μL extra media (LB with 2% glucose). A sterility control and a water barrier were also included. After a 24 h incubation at 37 °C, samples were washed thrice with sterile 20% MHB II. Equal parts of 20% MHB II and protein/peptide treatments (100 μL) were added to each well and incubated for 24 h at 37 °C to obtain a final 10%MHB II. Synthetic peptides were used at a final concentration of 4 μM, while recombinant proteins, designed as tetrameric HDPs, were used at a final concentration of 1 μM. Protein buffer was used as a negative control and mixed to 20% MHB media to be used as sterility controls. Wells were washed thrice with sterile 0.9% NaCl, treated with 100% methanol for 10 min to fix the bacteria, and stained with 1% crystal violet for 20 min. Three washes with distilled water were performed to remove the excess dye, and stained samples were dissolved with 70% EtOH. Samples were transferred to a new 96-well plate (100 μL/well) to read absorbance at 595 nm (LUMIstar^®^ Omega, v5.50R4, BMG Labtech, Ortenberg, Germany). The reduction in biofilm mass was calculated from triplicates with the same equation used for the BacTiterGlo bactericidal assay (Equation (1)).

### 2.6. Lipopolysaccharide Binding Assay

The ability of HPDs to bind LPS was evaluated using the fluorescent probe BIODIPY^®^ cadaverine (BC) (Invitrogen, Inchinnan, United Kingdom) and soluble LPS of *E. coli* O111:B4 (437627, Sigma-Aldrich, Madrid, Spain). Cadaverine specifically binds to LPS, and the conjugated fluorophore reports the displacement of the molecule as a shift in fluorescence. The LPS binding activity was assessed by measuring the displacement of BC at increasing concentrations of synthetic and recombinant HDPs prepared in 2-fold serial dilution in polystyrene 96-well plates with 10 mM HEPES buffer (pH 7.2). LPS and BC were added at a final concentration of 2.5 μg/mL and 2.5 μM, respectively, with HDP concentrations ranging from 7.5 to 0.007 μM. The following three controls were included: without LPS (NL), without peptide/protein (NP), and one with the synthetic peptide LL37 (synthesized and provided by the Systems Biology of Infection Lab, UAB), a well-characterized human antimicrobial peptide. The fluorescence was measured at 580 nm excitation and 620 nm emission wavelengths, both at a bandwidth of 5 nm and 30 flashes per well using a TECAN Spark Instrument (Tecan, Männedorf, Switzerland). Sample concentrations were transformed to the logarithm and data normalized to run from 0 to 100% to create a dose-response curve. These were fitted to a four-parameter logistic equation to calculate the half-maximal effective concentration (EC_50_); the concentration at which half of the protein is bound to LPS. The experiment was run in duplicates.

### 2.7. Dynamic Light Scattering (DLS)

The intensity size distribution of HDPs was assessed by dynamic light scattering (DLS) in a Zetasizer Pro Blue (Malvern Instruments Limited, Malvern, UK). HDP samples were prepared at 0.250 mg/mL in 20 mM HEPES buffer (pH 5.2) with or without a treatment with 10% SDS for 30 min at RT to dissociate any protein complexes. All were centrifuged at 15,000× *g* and 4 °C for 15 min before measurement. Samples were loaded in ZEN2112 3 mm quartz batch cuvettes, and scattered light was measured at 633 nm and 25 °C for three consecutive measurements. Volume size data were used to select the most representative population intensity values, which were subsequently represented. Polydispersity index (PDI) values were also displayed, providing protein size dispersion within the sample and their respective errors.

### 2.8. Secondary Structure of Peptides and Proteins

#### 2.8.1. Circular Dichroism Spectroscopy (CD)

Peptide and protein samples were dissolved in 20 mM HEPES at a concentration of 20 μM. They were then further diluted to a concentration of 6.7 μM with endotoxin-free distilled water or 5.4 μM with sodium dodecyl sulfate (SDS) micelles at a final concentration of 10 mM SDS to simulate a membrane-like environment [54,55]. CD spectra were recorded in the 190–260 nm range on a Jasco J-815 CD spectropolarimeter (Jasco, Easto, MD, USA) with a 3 mm quartz cuvette. OriginPro software (version 2022, OriginLab Corporation, Massachusetts, USA) was used to subtract the baseline signal from the buffer or SDS and to smooth data with the Svitzky–Golay method. Results were represented as molar mean residue ellipticity (ϑ_MRE_):

(2) $${\;[\theta ]}_{MRE}\;(deg\times {cm}^{2}\times {dmol}^{-1})=\frac{\theta \times 10^{6}}{l\times c\times n}$$

where *ϴ* is the ellipticity (*mdeg*); l is the cuvette pathlength (mm); *c* is the peptide/protein concentration (μM); and *n* is the number of peptide bonds = #aa − 1.

The CD spectra were analyzed with CDPro software (version 2021, N. Sreerama, [56]) to estimate the secondary structure fractions.

#### 2.8.2. ATR-FTIR Spectroscopy

An Alpha II FTIR spectrometer (Bruker, Bremen, Germany) with an attenuated total reflection (ATR) platinum accessory was used to study the amide I band of synthetic peptides and recombinant proteins. The measurement was conducted at the range of 1600–1700 cm^−1^, the amide I region, predominantly associated with the C=O stretching vibrations of the peptide backbone [57]. Each spectrum was acquired with a resolution of 4 cm^−1^ and averaged over 32 independent scans. Prior to measurement, all samples were dried to avoid the interference of water molecules, which absorb near 1640–1650 cm^−1^, overlapping with random coil and α-helix structures [58]. The OPUS MIR Tensor 27 software (Bruker, Bremen, Germany) was used to subtract the background, correct the baseline, and normalize the data. To resolve overlapping spectral components, second-derivative analysis was applied to the amide I region. Identified frequencies were subsequently modeled as overlapping Gaussian functions, from which the peak positions and areas were calculated using the PeakFit software package (Systat Software v4.12, San Jose, CA, USA).

This analysis revealed three deconvoluted peaks centered approximately at ~1630, ~1650, and ~1675 cm^−1^ in all samples.

## 3. Results

### 3.1. Protein Design and Physicochemical Characteristics

**Figure 1:**
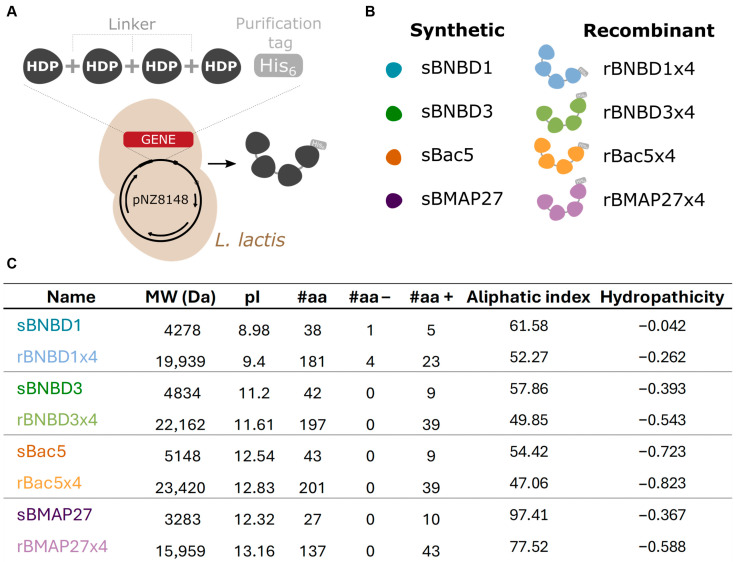
Synthetic peptides and recombinant protein characteristics. (**A**) Graphic representation of the design and production strategy of recombinant HDP-based proteins in L. lactis. (**B**) Scheme of the synthetic and recombinant versions of the HDPs used. (**C**) Physicochemical characteristics of synthetic HDPs and recombinant proteins estimated using ExPASy’s ProtParam server [59].

The potential of four bovine HDPs (Table 1), including two β-defensins (BNBD1 and BNBD3) and two cathelicidins (Bac5 and BMAP27), was compared in their synthetic and recombinant forms (Figure 1B), produced using *L. lactis*, a GRAS expression system (Figure 1A). HDPs with diverse characteristics were selected.

BNBD1 and BNBD3 share similar folding consisting of an α-helix and three antiparallel β-sheets (Supplementary Figure S1) but differ in amino acid composition and physicochemical properties (Figure 1C). BNBD1 has one anionic and five cationic residues, while BNBD3 has nine positively charged residues, showing a higher isoelectric point (pI). Additionally, BNBD3 (hydropathicity index −0.393) is more water-soluble than BNBD1 (hydropathicity index −0.042). Bac5 and BMAP27 are cathelicidins with major differences. Despite having similar pI, they differ in hydropathicity, aliphaticity, and primary structure (Table 1; Figure 1C). Bac5 is a linear cathelicidin enriched with 20 proline residues, being an amino acid rarely found in α-helical structures; whereas BMAP27 is cathelicidin, similar to human LL37, that acquires an α-helical when interacting with bacterial membranes (Supplementary Figure S1).

Peptides were chemically synthesized, while recombinant versions were produced in *L. lactis* as multidomain proteins containing four tandem copies of the same HDP (Figure 1A; Table S1 Supplementary Material). Key characteristics of both synthetic and recombinant HDPs have been summarized in Figure 1C. The recombinant forms have molecular weights and amino acid lengths approximately 4.7–5 times greater than their synthetic counterparts. They also have three additional positive charges because they were designed as tetramers linked by a 7-residue linker (GGSSRSS), which includes a cationic arginine (R), to facilitate an efficient recombinant expression (Supplementary Material Table S1).

### 3.2. Bioactivity Analysis

#### 3.2.1. Antimicrobial and Antibiofilm Activity

**Figure 2:**
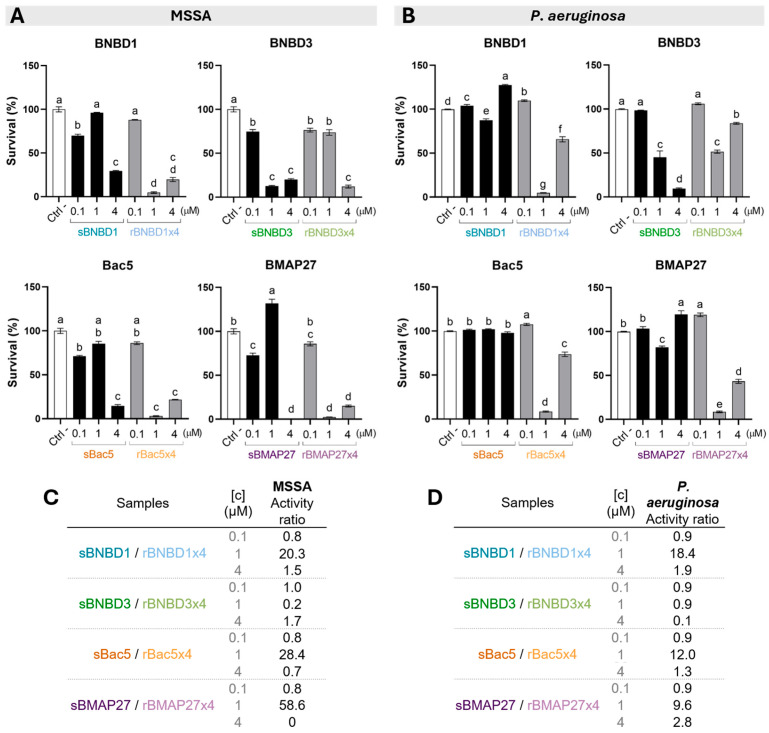
Antimicrobial activity of the synthetic peptides and recombinant proteins against (**A**) MSSA (Gram-positive) and (**B**) *P. aeruginosa* (Gram-negative). Ratio of bactericidal activity comparing synthetic and recombinant HDPs against (**C**) MSSA and (**D**) *P. aeruginosa*. Statistical differences calculated using a two-way ANOVA with the Tukey post hoc test (statistical differences determined by letters; *p* < 0.05).

**Figure 3:**
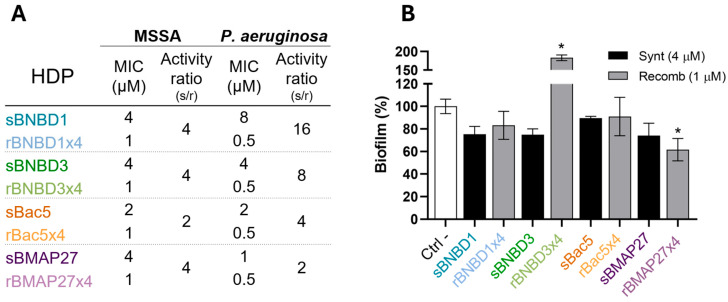
Bacteriostatic and antibiofilm activity determination. (**A**) MIC and bacteriostatic (MIC) activity ratio comparing synthetic and recombinant HDPs against MSSA and *P. aeruginosa*. (**B**) Antibiofilm assay against *P. aeruginosa*. Statistical differences calculated using a two-way ANOVA with Dunnett’s post hoc test to compare with the negative control (“*” = *p* < 0.005).

The antimicrobial activity of all molecules was evaluated against methicillin-sensitive *Staphylococcus aureus* (MSSA, Gram-positive) and *Pseudomonas aeruginosa* (Gram-negative) to assess if they had efficacy against the two bacterial groups. Multiple assays were used, including a bactericidal assay (Figure 2) and determination of the minimum inhibitory concentration (MIC) (Figure 3). In addition, the antibiofilm activity was assessed against *P. aeruginosa* (Figure 3).

The killing activity was evaluated by testing HDPs at 0.1, 1, and 4 μM concentrations. Although results revealed varying degrees of antimicrobial activity among the HDPs, the activity of recombinant version of three of the four peptides tested (BNBD1, Bac5, and BMPA27) was higher than the synthetic peptides (Figure 2). It is also worth noting that these three proteins were most effective at 1 μM against both the Gram-positive (Figure 2A) and the Gram-negative bacteria (Figure 2B). By contrast, the synthetic peptides demonstrated notable efficacy at 4 μM against *S. aureus* (Figure 2A) but had relatively lower or no activity against *P. aeruginosa* (Figure 2B). The survival rate ratio was calculated to compare bacteria treated with synthetic peptides to those of the recombinant forms. At 1 μM, the survival rate was 20–59% higher for MSSA (Figure 2C) and 9–18% for *P. aeruginosa* (Figure 2D) when bacteria were treated with synthetic peptides compared to their recombinant forms. At 0.1 and 4 μM, slight differences were observed (Figure 2C,D). However, BNBD3 exhibited the opposite trend: the synthetic was more active than its recombinant counterpart at 1 μM against *S. aureus* (Figure 2A,C) and at 4 μM against *P. aeruginosa* (Figure 2B,D).

The MIC values further supported these findings. Recombinant proteins showed lower MIC values and, thus, higher bacteriostatic activity than synthetic HDPs against both pathogenic bacteria (Figure 3A). All recombinant peptides were equally effective, with MIC values of 1 μM against MSSA and 0.5 μM against *P. aeruginosa*. The recombinants were four times more effective against MSSA than the synthetic peptides (MIC = 4 μM), except for Bac5, which was twice as effective (Figure 3A). However, MIC values varied among synthetic peptides against the Gram-negative bacteria, ranging from 1 to 8 μM, with sBMAP27 being the most active and sBNBD1 the least (Figure 3A). These were translated in recombinant rBMAP27x4 twice and rBac5x4 fourfold more active than the synthetic, but rBNBD3x4 8-times and rBNBD1x4 16-times more effective (Figure 3A).

The antibiofilm activity was relevant for the rBMAP27x4, the only one that significantly reduced the *P. aeruginosa* biofilm formation to almost 60% compared to the negative control (Figure 3B).

#### 3.2.2. Antiendotoxin Activity

**Figure 4:**
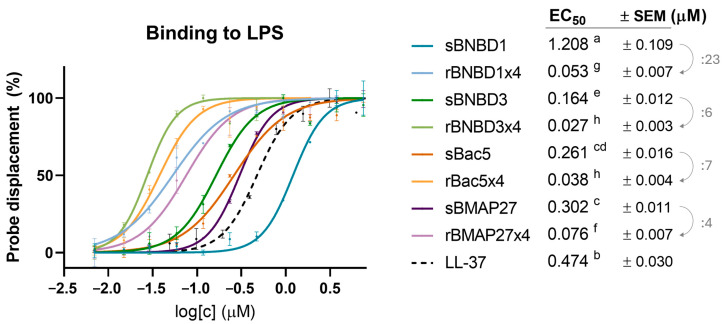
Binding of HDPs to soluble LPS. EC_50_ values are shown in concentration units (μM). A one-way ANOVA corrected with a Tukey multiple comparisons test was performed on logEC_50_ values, and statistical differences are denoted with letters (*p* < 0.05).

Another important mode of action of HDPs is the ability to bind to lipopolysaccharides (LPS) in the context of Gram-negative infections. This activity was assessed using a cadaverine fluorescent probe, which specifically binds to LPS, and followed its dissociation at increasing concentrations of HDPs. All HDPs, except sBNBD1, showed significantly better binding results to soluble LPS (low EC_50_ values) than the positive control of LL-37, a human cathelicidin known to bind this endotoxin (Figure 4) [60]. The efficacy of recombinant proteins was significantly greater, showing EC_50_ values between 4 and 23 times lower than the synthetic peptides (Figure 4). Recombinant rBac5x4 and rBNBD3x4 proved to be the best binding LPS, with EC_50_ values of 0.038 ± 0.004 μM and 0.027 ± 0.003 μM, respectively.

### 3.3. Structural Characterization and Conformational Analysis

#### 3.3.1. Size Distribution

**Figure 5:**
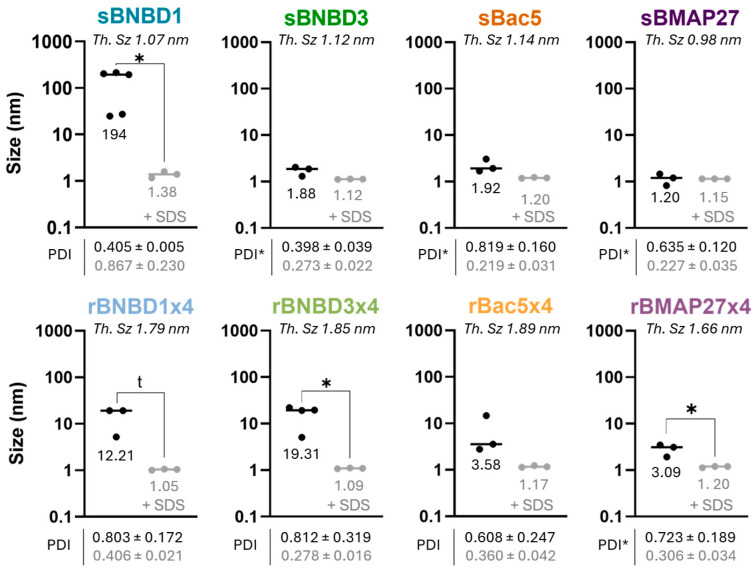
Size distribution in nm of synthetic and recombinant HDPs by DLS. Theoretical size estimated as Th. Sz = 0.066 ×*MW^1/3^ [61]. Intensity-weighted hydrodynamic diameter of synthetic and recombinant HDPs. Size values of HDPs in protein buffer alone represented as black dots and grey when treated with 10% SDS; median values are indicated. Lines indicate the median size of each sample. Polydispersity index values (PDI) are shown as mean value ± SD; in black for HDP alone and grey when treated with 10% SDS. Significant differences between +/− SDS were analyzed with unpaired *t*-tests and the Welch’s correction when SD were not equal. (*): *p*-value < 0.05; (t): *p*-value ≥ 0.0507 (tendency).

The size of HDPs was assessed using dynamic light scattering (DLS). Samples were analyzed in protein buffer to evaluate their protein conformation and oligomerization state; while SDS was added to disrupt non-covalent interactions, allowing us to study their monomeric form. The intensity-weighted size distributions revealed differences in particle size of synthetic and recombinant forms (Figure 5). In HEPES buffer, the sizes of the synthetic peptides ranged from 1.2 to 1.92 nm, except for sBNBD1, which aggregated (194 nm). However, the addition of SDS enabled the dissociation of sBNBD1 aggregates into 1.38 nm particles but did not significantly affect the other peptides (Figure 5). In contrast, the recombinant forms exhibited higher sizes, particularly rBNBD1x4 and rBNBD3x4, with size values of 12.21 and 19.31 nm, respectively. Treatment with SDS reduced the size of all recombinant forms to 1.20–1.05 nm, with statistically significant differences observed for rBNBD3x4 and rBMAP27x4, and a tendency for rBNBD1x4 (*p* = 0.0507).

#### 3.3.2. Protein Folding

**Figure 6:**
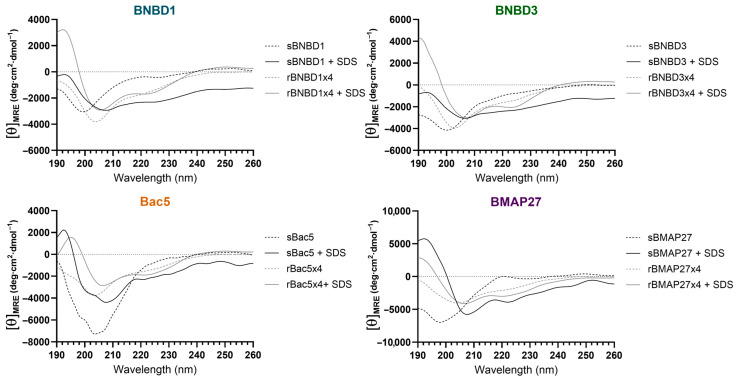
CD spectra of synthetic and recombinant HDPs. Discontinuous lines represent the CD spectra of samples in an aqueous environment, while continuous lines represent those in a membrane-like environment with SDS micelles (10 mM SDS).

**Figure 7:**
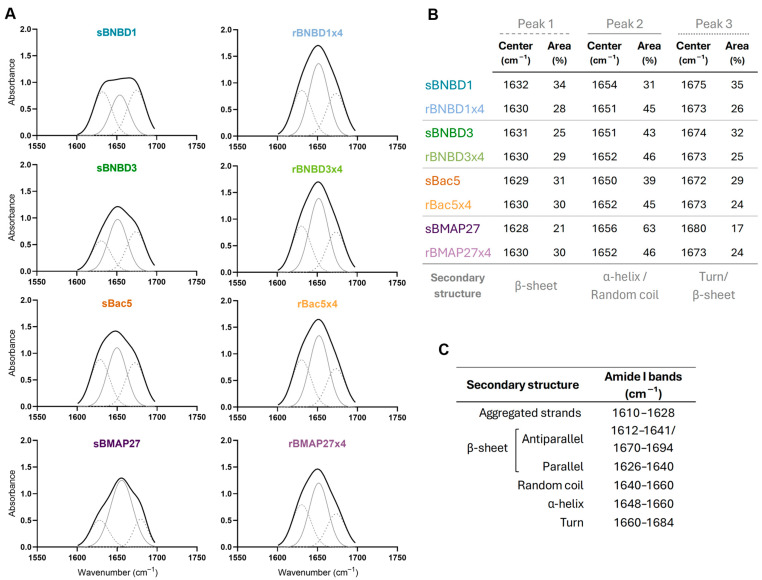
Analysis of the secondary structure content of synthetic and recombinant HDPs using FTIR-ATR. (**A**) The absorbance spectra in the amide I region (in black) and the three components obtained from the Fourier deconvolution of the FTIR spectra are represented: peak 1 (- -), peak 2 (—), and peak 3 (··). (**B**) Contribution of the secondary structure components indicating the area and center of each peak. (**C**) Summary table of typical amide I band assignments. [58,62,63,64,65].

The HDPs’ folding and secondary structures were analyzed using circular dichroism (CD) (Figure 6) and attenuated total reflectance–Fourier-transform infrared spectroscopy (ATR-FTIR) (Figure 7).

In an aqueous environment, all synthetic peptides exhibited CD spectra with strong minima below 200 nm, indicative of predominantly unordered conformations [66] (Figure 6 and Supplementary Table S2). In the membrane-like environment (SDS micelles), the CD spectra shifted toward features typical of α-helices, characterized by a maximum around 190–195 nm and two minima at 208 nm and 222 nm [66]. These signals were particularly intense in the recombinant variants, likely due to their multidomain structures (Supplementary Figure S2). Except for rBac5x4, these recombinant proteins already exhibited α-helical content even in aqueous solution, which became more pronounced in the presence of SDS micelles (Figure 6 and Supplementary Table S2), highlighting the conformational flexibility of all HDPs. However, the lack of combination of minimums at 206 and 218 nm observed in β-defensins peptides [67] suggests the absence of an αβ domain.

The ATR-FTIR spectra exhibited broad peaks centered around 1650 cm^−1^, indicating contributions from multiple secondary structures. Curve fitting and second derivative analysis revealed three distinct peaks at ~1630, ~1650, and ~1675 cm^−1^, associated with β-sheet structures, α-helix or random coil elements, and antiparallel β-sheets or β-turns, respectively ([58,62,63,64,65] summarized in Figure 7C). The relative area of each peak indicates each secondary structure’s contribution to the protein’s folding.

ATR-FTIR spectra varied among synthetic peptides but were consistent across recombinant proteins (Figure 7A,B). Specifically, recombinant proteins exhibited peaks at 1630, 1652 (rBNBD1x4 1651 cm^−1^), and 1673 cm^−1^ with similar relative areas of 28–30%, 45–46%, and 24–26%, respectively (Figure 7B). These data suggest a greater contribution of β-sheets in the structure of HDPs compared to what was inferred from CD analysis (Figure 6 and Supplementary Table S2). Still, both techniques consistently indicated α-helical content and suggest that the protein design used in recombinant HDPs could affect folding and structural stability.

## 4. Discussion

With the growing threat of antibiotic resistance, HDPs have emerged as a promising antimicrobial strategy owing to their broad-spectrum activity and multifunctionality [11,12]. However, despite their potential, challenges related to production, stability, and scalability have hindered clinical applications [29].

To address these limitations, we engineered recombinant tetrameric HDPs expressed in *L. lactis* and compared them with their synthetic linear monomers. Four bovine HDPs with different characteristics were selected: two β-defensins (BNBD1, BNBD3) and two cathelicidins (Bac5, and BMAP27) (Table 1). Previous studies comparing synthetic and recombinant HDPs reported either equivalent activity [35,52,53] or reduced efficacy in recombinant forms [68,69]. However, fusion carrier proteins such as thioredoxin were included in the recombinant forms reported in the literature when compared to synthetic ones. Thus, in our approach, we have designed recombinant HDP tetramers without the need for using any carrier protein. Our results revealed compelling advantages of recombinant tetramers over their synthetic monomeric counterparts, with enhanced biological activity and a more ordered structural profile.

Antimicrobial efficacy of synthetic and recombinant bovine peptides was evaluated through killing activity (Figure 2), MIC determination (Figure 3A), and antibiofilm activity (Figure 3B). Overall, recombinant tetramers not only retained but amplified their antimicrobial potency against MSSA (Gram-positive) (Figure 2C) and *P. aeruginosa* (Gram-negative) (Figure 2D). Notably, recombinant rBNBD1x4, rBac5x4, and rBMAP27x4 achieved significant bacterial killing of both pathogens at 1 μM, while synthetic versions required 4 μM to reach similar efficacy against MSSA (Figure 2A) and showed no activity against *P. aeruginosa* (Figure 2B). In the bactericidal assay, BNBD3 exhibited a behavior that differs from other recombinant HDPs, being more active in its synthetic form than in the recombinant one (Figure 2). However, when the MIC was assessed, we observed lower MIC values and consequently better bacteriostatic activity for all recombinant peptides, including rBNBD3x4 (Figure 3A).

This activity improvement of the recombinant forms when compared to the synthetic peptides suggested that the tetrameric format of the recombinant proteins could be related to these differences (Supplementary Figure S2). However, the increase in activity was not directly proportional to the number of peptide repeats. At 1 μM, recombinant HDPs exhibited a 10- to 59-fold increase in killing activity compared to their synthetic forms, except for BNBD3 (Figure 2). MIC values for recombinant β-defensins also improved significantly, especially against *P. aeruginosa* (Figure 3A). Furthermore, recombinant forms bound LPS more efficiently, with EC_50_ values an order of magnitude lower than those of their synthetic counterparts (Figure 4). Interestingly, rBMAP27x4 also exhibited antibiofilm activity against *P. aeruginosa*. Thus, altogether these findings suggest that the activity observed with the recombinant HDPs is not only a linear consequence of fusing four domains. These findings align with the current understanding that HDPs exhibit concentration-dependent effects, although not in a strictly linear manner [70,71,72,73]. For instance, increasing concentrations of Bac7 shift its mechanism of action from intracellular targeting to membrane lysis [74]. Moreover, some HDPs form oligomers or nanostructures, which are thought to play a critical role in their mechanism of action [75,76,77,78]. LL-37 tetramers, for example, seem to mediate membrane disruption through pore formation [75].

Recombinant HDPs were also more efficient than synthetic peptides binding LPS (Figure 4). However, recombinant rBMAP27, being the most active against the tested pathogens (Figure 2 and Figure 3A) and the only one capable of reducing the biofilm formed by *P. aeruginosa* (Figure 3B), had the weakest binding to LPS (Figure 4). These results suggested that electrostatic forces may not be the primary driver of binding LPS (negatively charged), since rBMAP27x4 was the most positively charged protein (Figure 1).

Activity differences between synthetic and recombinant HDPs could also be correlated with their assembly behavior. DLS measurements showed that recombinant proteins, showing higher activity, formed particles within the nanometer range (Figure 5). The formation of these nanoparticles with proteins comprising an N-terminal cationic peptide and a C-terminal His-tag has been previously reported [79,80,81,82]. Such constructs adopt a toroidal shape, with His-tags oriented inward and cationic peptides exposed on the surface [82,83]. In this study, beta-defensins BNBD1 and BNBD3 formed larger particles (19 nm and 12 nm, respectively) than cathelicidins Bac5 and BMAP27, indicating that β-defensins assemblies may consist of a greater number of molecules (Figure 5). Recombinant HDPs were fully disassembled upon 10% SDS, decreasing their size to values comparable to those found for the synthetic version (Figure 5). This self-assembly behavior may have a role in increasing protein activity and protecting recombinant HDPs from proteolytic degradation, compared to synthetic peptides, addressing a major limitation in the development of HDP-based therapeutics. In addition, the scalability of the recombinant production process makes this strategy a promising and cost-effective platform to produce HDPs for in vivo applications.

Furthermore, structural analyses (CD and ATR-FTIR) revealed that regardless of the production method, all HDPs exhibited structural flexibility, undergoing conformational changes from aqueous to hydrophobic environments (Figure 6 and Figure 7). The random coil content observed in the protein buffer was reduced when HDPs interacted with membrane-like structures (SDS micelles, Figure 6) or when water molecules were removed (Figure 7), suggesting a shift toward more structured forms. Synthetic peptides had a higher proportion of coil content and greater conformational flexibility (Figure 6). Recombinant tetramers exhibited remarkably similar CD and ATR-FTIR profiles, despite being based on different HDPs (Figure 6 and Figure 7). These findings suggest that multimeric architecture could promote intramolecular hydrogen bonding, favoring internal stabilization over interactions with surrounding water molecules. Our results indicated that both β-defensins (BNBD1 and BNBD3) exhibit conformational flexibility [84,85,86,87,88] and showed signs of β-sheet content, as suggested by ATR-FTIR (Figure 7). It is known that some β-defensins are more structurally stable than others, but all of them fold into an αβ domain stabilized by disulfide bonds [67,84,87,88,89,90]. However, the absence of a maximum at 230 nm in the CD spectrum, typically associated with disulfide bonds [67], suggested that proper folding into the canonical αβ domain may have been compromised. This effect could be more pronounced in the recombinant variants, potentially explaining the lower activity observed for rBNBD3x4. In this context, it is important to remark that the disulfide bonds are considered important for β-defensin stability [68,91], but previous studies have shown that correct folding and activity can occur even in their absence [87,90,92].

Furthermore, Bac5, despite its high proline content, showed structural flexibility and a shift toward α-helical structure in membrane-like conditions (10 mM SDS) when analyzed by CD (Figure 6). This conformational adaptability may contribute to the enhanced activity observed in rBac5x4. The high activity and antibiofilm effect of rBMAP27x4 likely correlate with its ability to adopt α-helical structures in membrane-mimetic environments, consistent with known pore-forming mechanisms. Overall, these findings, together with previous studies, highlighted that structural stabilization—particularly proper folding and environment-induced conformational changes—is a key determinant for antimicrobial activity and the mechanism of action of AMPs [93]. These findings support the idea that improved activity in recombinant HDPs, compared to their synthetic counterparts, is due not only to a higher local concentration of functional domains, but also to structural conformations that favor peptide–membrane interactions.

## 5. Conclusions

The recombinant strategy described in this work is a promising alternative for producing highly active HDPs. Linking four copies of the same HDP influences structural organization and amplifies bioactivity beyond a simple dose-dependent effect. Recombinant antimicrobial proteins exhibited stronger antimicrobial activity against both MSSA and *P. aeruginosa* compared to monomeric synthetic counterparts. Additionally, these recombinant tetramers have also improved LPS binding and antibiofilm activity positioning them as a viable and efficient production strategy beyond conventional chemical synthesis.

## Figures and Tables

**Table 1:** Primary structure of bovine HDPs.

Class	Name	Sequence
β-Defensins	BNBD1	DFASC_1_HTNGGIC_2_LPNRC_3_PGHMIQIGIC_4_FRPRVKC_5_C_6_RSW
	BNBD3	QGVRNHVTC_1_RINRGFC_2_VPIRC_3_PGRTRQIGTC_4_FGPRIKC_5_C_6_RSW
Cathelicidins	Bac5	RFRPPIRRPPIRPPFYPPFRPPIRPPIFPPIRPPFRPPLGPFP
	BMAP27	GRFKRFRKKFKKLFKKLSPVIPLLHLG

Numbers in β-defensin sequences denote the six cysteines characteristic of these HDPs that have a Cys pairing: Cys1–Cys5, Cys2–Cys4, and Cys3–Cys6, typical of vertebrate β-defensins.

## Data Availability

The raw data supporting the conclusions of this article will be made available by the authors on request.

## Supplementary Materials

The following supporting information can be downloaded at: https://www.mdpi.com/article/10.3390/biom15070980/s1, Figure S1: Three-dimensional structure of BNBD1 (a), BNBD3 (b), Bac5 segment 131-173 (c) and BMAP27 segment 132-158 (d) according to their PDB. Tertiary structures are represented using PyMOL software (v3.0.2, Schrödinger Inc., New York, USA) where α-helix is red, β-strand is in yellow, and loop is in green; Figure S2: Three-dimensional structure of rBNBD1x4 (a), rBNBD3x4 (b), rBac5x4 segment (c) and rBMAP27x4 (d) as predicted by AlphaFold3 [94]. Tertiary structures are represented using PyMOL software. α-helix in red, β-strands in yellow, loops in green, and the linker (GGSSRSS) in violet; Table S1: Sequence of the recombinant proteins rBNBD1x4, rBNBD3x4, rBac5x4, and rBMAP27x4. GGSSRSS was used as a linker between HDP domains, and a 6xHis-tag was added at the C-terminus for protein purification purposes; Table S2: Analysis of CD spectra and prediction of secondary structure using the CONTIN/LL method with the CDPro software package.

## Abbreviations

The following abbreviations are used in this manuscript:

HDPs	host defense peptides
LPS	lipopolysaccharides
SPPS	solid-phase peptide synthesis
PTMs	post-translational modifications
GRAS	generally regarded as safe
BNBD1	bovine neutrophil β-defensin 1
BNBD3	bovine neutrophil β-defensin 3
Bac5	bactenecin 5
BMAP27	bovine myeloid antibacterial peptide 27
MSSA	methicillin-sensitive *Staphylococcus aureus*
BHI	brain heart infusion
LB	Luria-Bertani
IMAC	immobilized metal affinity chromatography
MHB	Mueller Hinton Broth
MIC	minimum inhibitory concentration
DLS	dynamic light scattering
ATR-FTIR	attenuated total reflectance–Fourier-transform infrared spectroscopy

## References

[1] Ikuta K.S., Swetschinski L.R., Robles Aguilar G., Sharara F., Mestrovic T., Gray A.P., Davis Weaver N., Wool E.E., Han C., Gershberg Hayoon A. (2022). Global Mortality Associated with 33 Bacterial Pathogens in 2019: A Systematic Analysis for the Global Burden of Disease Study 2019. Lancet.

[2] Naghavi M., Mestrovic T., Gray A., Gershberg Hayoon A., Swetschinski L.R., Robles Aguilar G., Davis Weaver N., Ikuta K.S., Chung E., Wool E.E. (2024). Global Burden Associated with 85 Pathogens in 2019: A Systematic Analysis for the Global Burden of Disease Study 2019. Lancet Infect. Dis..

[3] Cella E., Giovanetti M., Benedetti F., Scarpa F., Johnston C., Borsetti A., Ceccarelli G., Azarian T., Zella D., Ciccozzi M. (2023). Joining Forces against Antibiotic Resistance: The One Health Solution. Pathogens.

[4] Robi D.T., Mossie T., Temteme S. (2024). A Comprehensive Review of the Common Bacterial Infections in Dairy Calves and Advanced Strategies for Health Management. Vet. Med. Res. Rep..

[5] Serra-Burriel M., Keys M., Campillo-Artero C., Agodi A., Barchitta M., Gikas A., Palos C., López-Casasnovas G. (2020). Impact of Multi-Drug Resistant Bacteria on Economic and Clinical Outcomes of Healthcare-Associated Infections in Adults: Systematic Review and Meta-Analysis. PLoS ONE.

[6] Cloeckaert A., Kuchler K. (2020). Grand Challenges in Infectious Diseases: Are We Prepared for Worst-Case Scenarios?. Front. Microbiol..

[7] Arnold K.E., Laing G., McMahon B.J., Fanning S., Stekel D.J., Pahl O., Coyne L., Latham S.M., McIntyre K.M. (2024). The Need for One Health Systems-Thinking Approaches to Understand Multiscale Dissemination of Antimicrobial Resistance. Lancet Planet. Heal..

[8] Mahlapuu M., Håkansson J., Ringstad L., Björn C. (2016). Antimicrobial Peptides: An Emerging Category of Therapeutic Agents. Front. Cell. Infect. Microbiol..

[9] Kang H.K., Kim C., Seo C.H., Park Y. (2017). The Therapeutic Applications of Antimicrobial Peptides (AMPs): A Patent Review. J. Microbiol..

[10] Van Dijk A., Hedegaard C.J., Haagsman H.P., Heegaard P.M.H. (2018). The Potential for Immunoglobulins and Host Defense Peptides (HDPs) to Reduce the Use of Antibiotics in Animal Production. Vet. Res..

[11] Mookherjee N., Anderson M.A., Haagsman H.P., Davidson D.J. (2020). Antimicrobial Host Defence Peptides: Functions and Clinical Potential. Nat. Rev. Drug Discov..

[12] Drayton M., Deisinger J.P., Ludwig K.C., Raheem N., Müller A., Schneider T., Straus S.K. (2021). Host Defense Peptides: Dual Antimicrobial and Immunomodulatory Action. Int. J. Mol. Sci..

[13] Graf M., Mardirossian M., Nguyen F., Seefeldt A.C., Guichard G., Scocchi M., Innis C.A., Wilson D.N. (2017). Proline-Rich Antimicrobial Peptides Targeting Protein Synthesis. Nat. Prod. Rep..

[14] Ho Y.H., Shah P., Chen Y.W., Chen C.S. (2016). Systematic Analysis of Intracellular-Targeting Antimicrobial Peptides, Bactenecin 7, Hybrid of Pleurocidin and Dermaseptin, Proline-Arginine-Rich Peptide, and Lactoferricin b, by Using Escherichia Coli Proteome Microarrays. Mol. Cell. Proteom..

[15] Raheem N., Straus S.K. (2019). Mechanisms of Action for Antimicrobial Peptides With Antibacterial and Antibiofilm Functions. Front. Microbiol..

[16] Martin L., van Meegern A., Doemming S., Schuerholz T. (2015). Antimicrobial Peptides in Human Sepsis. Front. Immunol..

[17] Rosenfeld Y., Shai Y. (2006). Lipopolysaccharide (Endotoxin)-Host Defense Antibacterial Peptides Interactions: Role in Bacterial Resistance and Prevention of Sepsis. Biochim. Biophys. Acta—Biomembr..

[18] Martell E.M., González-Garcia M., Ständker L., Otero-González A.J. (2021). Host Defense Peptides as Immunomodulators: The Other Side of the Coin. Peptides.

[19] Cai J., Li X., Du H., Jiang C., Xu S., Cao Y. (2020). Immunomodulatory Significance of Natural Peptides in Mammalians: Promising Agents for Medical Application. Immunobiology.

[20] van der Does A.M., Hiemstra P.S., Mookherjee N., Matsuzaki K. (2019). Antimicrobial Host Defence Peptides: Immunomodulatory Functions and Translational Prospects. Antimicrobial Peptides. Advances in Experimental Medicine and Biology.

[21] Gill I., López-Fandiño R., Jorba X., Vulfson E.N. (1996). Biologically Active Peptides and Enzymatic Approaches to Their Production. Enzym. Microb. Technol..

[22] Münzker L., Oddo A., Hansen P.R. (2017). Chemical Synthesis of Antimicrobial Peptides. Methods Mol. Biol..

[23] Kent S.B.H. (2009). Total Chemical Synthesis of Proteins. Chem. Soc. Rev..

[24] Zompra A.A., Galanis A.S., Werbitzky O., Albericio F. (2009). Manufacturing Peptides as Active Pharmaceutical Ingredients. Future Med. Chem..

[25] Pedersen S.L., Tofteng A.P., Malik L., Jensen K.J. (2012). Microwave Heating in Solid-Phase Peptide Synthesis. Chem. Soc. Rev..

[26] Skalska J., Andrade V.M., Cena G.L., Harvey P.J., Gaspar D.M.D., Mello É.O., Henriques S.T., Valle J., Gomes V.M., Conceição K. (2020). Synthesis, Structure, and Activity of the Antifungal Plant Defensin Pv D 1. J. Med. Chem..

[27] Gaglione R., Pane K., Dell’Olmo E., Cafaro V., Pizzo E., Olivieri G., Notomista E., Arciello A. (2019). Cost-Effective Production of Recombinant Peptides in *Escherichia coli*. N. Biotechnol..

[28] Jayakrishnan A., Wan Rosli W.R., Tahir A.R.M., Razak F.S.A., Kee P.E., Ng H.S., Chew Y.-L., Lee S.-K., Ramasamy M., Tan C.S. (2024). Evolving Paradigms of Recombinant Protein Production in Pharmaceutical Industry: A Rigorous Review. Sci.

[29] Boto A., De La Lastra J.M.P., González C.C. (2018). The Road from Host-Defense Peptides to a New Generation of Antimicrobial Drugs. Molecules.

[30] Hilchie A.L., Wuerth K., Hancock R.E.W. (2013). Immune Modulation by Multifaceted Cationic Host Defense (Antimicrobial) Peptides. Nat. Chem. Biol..

[31] Kang J., Zhao D., Lyu Y., Tian L., Yin X., Yang L., Teng K., Zhou X. (2014). Antimycobacterial Activity of Pichia Pastoris-Derived Mature Bovine Neutrophil β-Defensins 5. Eur. J. Clin. Microbiol. Infect. Dis..

[32] Choi H.J., Seo M.J., Lee J.C., Cheigh C.I., Park H., Ahn C., Pyun Y.R. (2005). Heterologous Expression of Human β-Defensin-1 in Bacteriocin-Producing Lactococcus Lactis. J. Microbiol. Biotechnol..

[33] Roca-Pinilla R., Lisowski L., Arís A., Garcia-Fruitós E. (2022). The Future of Recombinant Host Defense Peptides. Microb. Cell Fact..

[34] Deo S., Turton K.L., Kainth T., Kumar A., Wieden H.-J. (2022). Strategies for Improving Antimicrobial Peptide Production. Biotechnol. Adv..

[35] Clement H., Flores V., Diego-Garcia E., Corrales-Garcia L., Villegas E., Corzo G. (2015). A Comparison between the Recombinant Expression and Chemical Synthesis of a Short Cysteine-Rich Insecticidal Spider Peptide. J. Venom. Anim. Toxins Incl. Trop. Dis..

[36] Silva O.N., Mulder K.C., Barbosa A.A., Otero-Gonzalez A.J., Lopez-Abarrategui C., Rezende T.M.B., Dias S.C., Franco O.L. (2011). Exploring the Pharmacological Potential of Promiscuous Host-Defense Peptides: From Natural Screenings to Biotechnological Applications. Front. Microbiol..

[37] Bommarius B., Jenssen H., Elliott M., Kindrachuk J., Pasupuleti M., Gieren H., Jaeger K.-E., Hancock R.E.W., Kalman D. (2010). Cost-Effective Expression and Purification of Antimicrobial and Host Defense Peptides in Escherichia Coli. Peptides.

[38] Li Y. (2011). Recombinant Production of Antimicrobial Peptides in Escherichia Coli: A Review. Protein Expr. Purif..

[39] Perez-Perez D.A., Villanueva-Ramirez T.d.J., Hernandez-Pedraza A.E., Casillas-Vega N.G., Gonzalez-Barranco P., Zarate X. (2021). The Small Metal-Binding Protein Smbp Simplifies the Recombinant Expression and Purification of the Antimicrobial Peptide Ll-37. Antibiotics.

[40] Panteleev P.V., Bolosov I.A., Kalashnikov A.À., Kokryakov V.N., Shamova O.V., Emelianova A.A., Balandin S.V., Ovchinnikova T.V. (2018). Combined Antibacterial Effects of Goat Cathelicidins with Different Mechanisms of Action. Front. Microbiol..

[41] Wright O., Yoshimi T., Tunnacliffe A. (2012). Recombinant Production of Cathelicidin-Derived Antimicrobial Peptides in Escherichia Coli Using an Inducible Autocleaving Enzyme Tag. N. Biotechnol..

[42] van der Merwe J., Prysliak T., Gerdts V., Perez-Casal J. (2011). Protein Chimeras Containing the Mycoplasma Bovis GAPDH Protein and Bovine Host-Defence Peptides Retain the Properties of the Individual Components. Microb. Pathog..

[43] López-Cano A., Martínez-Miguel M., Guasch J., Ratera I., Arís A., Garcia-Fruitós E. (2022). Exploring the Impact of the Recombinant Escherichia Coli Strain on Defensins Antimicrobial Activity: BL21 versus Origami Strain. Microb. Cell Fact..

[44] Xin A., Zhao Y., Yu H., Shi H., Liu H., Diao H., Zhang Y. (2014). Soluble Fusion Expression, Characterization and Localization of Human β-Defensin 6. Mol. Med. Rep..

[45] Wu J., Wang C., He H., Hu G., Yang H., Gao Y., Zhong J. (2011). Molecular Analysis and Recombinant Expression of Bovine Neutrophil β-Defensin 12 and Its Antimicrobial Activity. Mol. Biol. Rep..

[46] Ojima-Kato T. (2025). Advances in Recombinant Protein Production in Microorganisms and Functional Peptide Tags. Biosci. Biotechnol. Biochem..

[47] Morello E., Bermúdez-Humarán L.G., Llull D., Solé V., Miraglio N., Langella P., Poquet I. (2008). Lactococcus Lactis, an Efficient Cell Factory for Recombinant Protein Production and Secretion. J. Mol. Microbiol. Biotechnol..

[48] Cano-Garrido O., Rueda F.L., Sànchez-García L., Ruiz-Ávila L., Bosser R., Villaverde A., García-Fruitós E. (2014). Expanding the Recombinant Protein Quality in Lactococcus Lactis. Microb. Cell Fact..

[49] Zhang H., Dong M., Xu H., Li H., Zheng A., Sun G., Jin W. (2024). Recombinant Lactococcus Lactis Expressing Human LL-37 Prevents Deaths from Viral Infections in Piglets and Chicken. Probiotics Antimicrob. Proteins.

[50] Baltà-Foix R., Garcia-Fruitós E., Arís A. (2024). Time to Consider Ruling out Inclusion Bodies Denaturing Protocols for Spontaneous Solubilization of Biologically Active Proteins. Sci. Rep..

[51] Wang T., Wang Z., Mi J., Wang W., Li K., Qi X., Gao Y., Gao L., Liu C., Zhang Y. (2021). Recombinant Avian β-Defensin Produced by Food-Grade Lactococcus as a Novel and Potent Immunological Enhancer Adjuvant for Avian Vaccine. Probiotics Antimicrob. Proteins.

[52] Harder J., Bartels J., Christophers E., Schröder J.-M. (2001). Isolation and Characterization of Human B-Defensin-3, a Novel Human Inducible Peptide Antibiotic. J. Biol. Chem..

[53] Morin K.M., Arcidiacono S., Beckwitt R., Mello C.M. (2006). Recombinant Expression of Indolicidin Concatamers in Escherichia Coli. Appl. Microbiol. Biotechnol..

[54] Perinelli D.R., Cespi M., Lorusso N., Palmieri G.F., Bonacucina G., Blasi P. (2020). Surfactant Self-Assembling and Critical Micelle Concentration: One Approach Fits All?. Langmuir.

[55] Bello-Madruga R., Sandín D., Valle J., Gómez J., Comas L., Larrosa M.N., González-López J.J., Jiménez M.Á., Andreu D., Torrent M. (2025). Mining the Heparinome for Cryptic Antimicrobial Peptides That Selectively Kill Gram-Negative Bacteria. Mol. Syst. Biol..

[56] Sreerama N., Woody R.W. (2000). Estimation of Protein Secondary Structure from Circular Dichroism Spectra: Comparison of CONTIN, SELCON, and CDSSTR Methods with an Expanded Reference Set. Anal. Biochem..

[57] Pribic R., Vanstokkum I.H.M., Chapman D., Haris P.I., Bloemendal M. (1993). Protein Secondary Structure from Fourier Transform Infrared and/or Circular Dichroism Spectra. Anal. Biochem..

[58] Tatulian S.A. (2013). Structural Characterization of Membrane Proteins and Peptides by FTIR and ATR-FTIR Spectroscopy. Methods Mol. Biol..

[59] Gasteiger E., Hoogland C., Gattiker A., Duvaud S., Wilkins M.R., Appel R.D., Bairoch A., Walker J.M. (2005). Protein Identification and Analysis Tools on the ExPASy Server. The Proteomics Protocols Handbook. Springer Protocols Handbook.

[60] Zeth K., Sancho-Vaello E. (2021). Structural Plasticity of LL-37 Indicates Elaborate Functional Adaptation Mechanisms to Bacterial Target Structures. Int. J. Mol. Sci..

[61] Erickson H.P. (2009). Size and Shape of Protein Molecules at the Nanometer Level Determined by Sedimentation, Gel Filtration, and Electron Microscopy. Biol. Proced. Online.

[62] Susi H., Byler D.M. (1986). Resolution-Enhanced Fourier Transform Infrared Spectroscopy of Enzymes. Methods in Enzymology.

[63] Goormaghtigh E., Cabiaux V., Ruysschaert J. (1990). Secondary Structure and Dosage of Soluble and Membrane Proteins by Attenuated Total Reflection Fourier-transform Infrared Spectroscopy on Hydrated Films. Eur. J. Biochem..

[64] Jackson M., Mantsch H.H. (1995). The Use and Misuse of FTIR Spectroscopy in the Determination of Protein Structure. Crit. Rev. Biochem. Mol. Biol..

[65] Yan Z., Li Q., Zhang P. (2017). Soy Protein Isolate and Glycerol Hydrogen Bonding Using Two-Dimensional Correlation (2D-COS) Attenuated Total Reflection Fourier Transform Infrared (ATR FT-IR) Spectroscopy. Appl. Spectrosc..

[66] Rogers D.M., Jasim S.B., Dyer N.T., Auvray F., Réfrégiers M., Hirst J.D. (2019). Electronic Circular Dichroism Spectroscopy of Proteins. Chem.

[67] Bello-Madruga R., Valle J., Jiménez M.Á., Torrent M., Montero-Alejo V., Andreu D. (2023). The C-Terminus of Panusin, a Lobster β-Defensin, Is Crucial for Optimal Antimicrobial Activity and Serum Stability. Pharmaceutics.

[68] Yang M., Zhang C., Zhang X., Zhang M.Z., Rottinghaus G.E., Zhang S. (2016). Structure-Function Analysis of Avian β-Defensin-6 and β-Defensin-12: Role of Charge and Disulfide Bridges. BMC Microbiol..

[69] Zhao L., Yang M., Zhang M., Zhang S. (2014). Expression, Purification, and in Vitro Comparative Characterization of Avian Beta-Defensin-2,-6, and-12. Avian Dis..

[70] Bonucci A., Balducci E., Pistolesi S., Pogni R. (2013). The Defensin–Lipid Interaction: Insights on the Binding States of the Human Antimicrobial Peptide HNP-1 to Model Bacterial Membranes. Biochim. Biophys. Acta—Biomembr..

[71] Sani M.-A., Separovic F. (2016). How Membrane-Active Peptides Get into Lipid Membranes. Acc. Chem. Res..

[72] Benincasa M., Lagatolla C., Dolzani L., Milan A., Pacor S., Liut G., Tossi A., Cescutti P., Rizzo R. (2016). Biofilms from Klebsiella Pneumoniae: Matrix Polysaccharide Structure and Interactions with Antimicrobial Peptides. Microorganisms.

[73] Xia R., Xiao H., Xu M., Hou L., Han Y., Zhou Z. (2024). Insight into the Inhibitory Activity and Mechanism of Bovine Cathelicidin BMAP 27 against Salmonella Typhimurium. Microb. Pathog..

[74] Podda E., Benincasa M., Pacor S., Micali F., Mattiuzzo M., Gennaro R., Scocchi M. (2006). Dual Mode of Action of Bac7, a Proline-Rich Antibacterial Peptide. Biochim. Biophys. Acta—Gen. Subj..

[75] Sancho-Vaello E., Gil-Carton D., François P., Bonetti E.J., Kreir M., Pothula K.R., Kleinekathöfer U., Zeth K. (2020). The Structure of the Antimicrobial Human Cathelicidin LL-37 Shows Oligomerization and Channel Formation in the Presence of Membrane Mimics. Sci. Rep..

[76] Sancho-Vaello E., François P., Bonetti E.J., Lilie H., Finger S., Gil-Ortiz F., Gil-Carton D., Zeth K. (2017). Structural Remodeling and Oligomerization of Human Cathelicidin on Membranes Suggest Fibril-like Structures as Active Species. Sci. Rep..

[77] Wommack A.J., Robson S.A., Wanniarachchi Y.A., Wan A., Turner C.J., Wagner G., Nolan E.M. (2012). NMR Solution Structure and Condition-Dependent Oligomerization of the Antimicrobial Peptide Human Defensin 5. Biochemistry.

[78] Chairatana P., Chiang I.L., Nolan E.M. (2017). Human α-Defensin 6 Self-Assembly Prevents Adhesion and Suppresses Virulence Traits of Candida Albicans. Biochemistry.

[79] Unzueta U., Ferrer-Miralles N., Cedano J., Zikung X., Pesarrodona M., Saccardo P., García-Fruitós E., Domingo-Espín J., Kumar P., Gupta K.C. (2012). Non-Amyloidogenic Peptide Tags for the Regulatable Self-Assembling of Protein-Only Nanoparticles. Biomaterials.

[80] Serna N., Sánchez J.M., Unzueta U., Sánchez-Garcia L., Sánchez-Chardi A., Mangues R., Vázquez E., Villaverde A. (2019). Recruiting Potent Membrane Penetrability in Tumor Cell-Targeted Protein-Only Nanoparticles. Nanotechnology.

[81] López-Laguna H., Unzueta U., Conchillo-Solé O., Sánchez-Chardi A., Pesarrodona M., Cano-Garrido O., Voltà E., Sánchez-García L., Serna N., Saccardo P. (2019). Assembly of Histidine-Rich Protein Materials Controlled through Divalent Cations. Acta Biomater..

[82] Céspedes M.V., Unzueta U., Tatkiewicz W., Sánchez-Chardi A., Conchillo-Solé O., Álamo P., Xu Z., Casanova I., Corchero J.L., Pesarrodona M. (2014). In Vivo Architectonic Stability of Fully de Novo Designed Protein-Only Nanoparticles. ACS Nano.

[83] Serna N., Sánchez-García L., Sánchez-Chardi A., Unzueta U., Roldán M., Mangues R., Vázquez E., Villaverde A. (2017). Protein-Only, Antimicrobial Peptide-Containing Recombinant Nanoparticles with Inherent Built-in Antibacterial Activity. Acta Biomater..

[84] Boniotto M., Antcheva N., Zelezetsky I., Tossi A., Palumbo V., Verga Falzacappa M.V., Sgubin S., Braida L., Amoroso A., Crovella S. (2003). A Study of Host Defence Peptide β-Defensin 3 in Primates. Biochem. J..

[85] Wu Z., Hoover D.M., Yang D., Boulègue C., Santamaria F., Oppenheim J.J., Lubkowski J., Lu W. (2003). Engineering Disulfide Bridges to Dissect Antimicrobial and Chemotactic Activities of Human β-Defensin 3. Proc. Natl. Acad. Sci. USA.

[86] Diao H., Guo C., Lin D., Zhang Y. (2007). Intein-Mediated Expression Is an Effective Approach in the Study of β-Defensins. Biochem. Biophys. Res. Commun..

[87] Liu S., Zhou L., Li J., Suresh A., Verma C., Foo Y.H., Yap E.P.H., Tan D.T.H., Beuerman R.W. (2008). Linear Analogues of Human β-Defensin 3: Concepts for Design of Antimicrobial Peptides with Reduced Cytotoxicity to Mammalian Cells. ChemBioChem.

[88] Morgera F., Antcheva N., Pacor S., Quaroni L., Berti F., Vaccari L., Tossi A. (2008). Structuring and Interactions of Human β-Defensins 2 and 3 with Model Membranes. J. Pept. Sci..

[89] Antcheva N., Boniotto M., Zelezetsky I., Pacor S., Falzacappa M.V.V., Crovella S., Tossi A. (2004). Effects of Positively Selected Sequence Variations in Human and Macaca Fascicularis β-Defensins 2 on Antimicrobial Activity. Antimicrob. Agents Chemother..

[90] Mandal M., Jagannadham M.V., Nagaraj R. (2002). Antibacterial Activities and Conformations of Bovine β-Defensin BNBD-12 and Analogs:Structural and Disulfide Bridge Requirements for Activity. Peptides.

[91] Krishnakumari V., Sharadadevi A., Singh S., Nagaraj R. (2003). Single Disulfide and Linear Analogues Corresponding to the Carboxy-Terminal Segment of Bovine β-Defensin-2: Effects of Introducing the β-Hairpin Nucleating Sequence D-Pro-Gly on Antibacterial Activity and Biophysical Properties. Biochemistry.

[92] Klüver E., Schulz-Maronde S., Scheid S., Meyer B., Forssmann W.G., Adermann K. (2005). Structure-Activity Relation of Human β-Defensin 3: Influence of Disulfide Bonds and Cysteine Substitution on Antimicrobial Activity and Cytotoxicity. Biochemistry.

[93] Bello-Madruga R., Torrent Burgas M. (2024). The Limits of Prediction: Why Intrinsically Disordered Regions Challenge Our Understanding of Antimicrobial Peptides. Comput. Struct. Biotechnol. J..

[94] Abramson J., Adler J., Dunger J., Evans R., Green T., Pritzel A., Ronneberger O., Willmore L., Ballard A.J., Bambrick J. (2024). Accurate Structure Prediction of Biomolecular Interactions with AlphaFold 3. Nature.

