# Supplementary material for: Evaluating Host Defense Peptides: A Comparative Analysis of Synthetic Peptides and Recombinant Concatemers
Source: Biomolecules. 2025 Jul 8;15(7):980. doi: 10.3390/biom15070980 (PMC12292752; doi:10.3390/biom15070980)
Supplement: Supplementary file 1 [file biomolecules-15-00980-s001.zip › biomolecules-3713380-supplementary.pdf]

Supplementary Materials

Supplementary Table S1: Sequence of the recombinant proteins rBNBD1x4, rBNBD3x4, rBac5x4, and rBMAP27x4. GGSSRSS was used as a linker between HDP domains, and a 6xHis-tag was added at the C-terminus for protein purification purposes.

Name	Sequence
rBNBD1x4	MADFASCHTNGGICLPNRCPGHMIQIGICFRPRVKCCRSWGGSSRSSDFASCHTNGGICLPNRCPGHMIQIGICFRPRVKCCRSWGGSSRSSDFASCHTNGGICLPNRCPGHMIQIGICFRPRVKCCRSWGGSSRSSDFASCHTNGGICLPNRCPGHMIQIGICFRPRVKCCRSW*HHHHHH*
rBNBD3x4	MAQGVRNHVTCRINRGFCVPIRCPGRTRQIGTCFGPRIKCCRSWGGSSRSSQGVRNHVTCRINRGFCVPIRCPGRTRQIGTCFGPRIKCCRSWGGSSRSSQGVRNHVTCRINRGFCVPIRCPGRTRQIGTCFGPRIKCCRSWGGSSRSSQGVRNHVTCRINRGFCVPIRCPGRTRQIGTCFGPRIKCCRSW*HHHHHH*
rBac5x4	MARFRPPIRRPPIRPPFYPPFRPPIRPPIFPPIRPPFRPPLGPFPGGSSRSSRFRPPIRRPPIRPPFYPPFRPPIRPPIFPPIRPPFRPPLGPFPGGSSRSSRFRPPIRRPPIRPPFYPPFRPPIRPPIFPPIRPPFRPPLGPFPGGSSRSSRFRPPIRRPPIRPPFYPPFRPPIRPPIFPPIRPPFRPPLGPFP*HHHHHH*
rBMAP27x4	MAGRFKRFRKKFKKLFKKLSPVIPLLHLGGGSSRSSGRFKRFRKKFKKLFKKLSPVIPLLHLGGGSSRSSGRFKRFRKKFKKLFKKLSPVIPLLHLGGGSSRSSGRFKRFRKKFKKLFKKLSPVIPLLHLG*HHHHHH*

Supplementary Table S2: Analysis of CD spectra and prediction of secondary structure using the CONTIN/LL method with the CDPro software package.

		Prediction of secondary structure (%)							
		sBNBD1	rBNBD1x4	sBNBD3	rBNBD3x4	sBac5	rBac5x4	sBMAP27	rBMAP27x4
Prot buffer	α-helix	10	44	23	40	24	44	6	44
	β-sheet	19	1	6	5	17	4	31	5
	Turn	24	27	25	31	23	30	23	23
	Unordered	47	28	46	24	36	22	40	28
	3º Structure	*uo*	*a+b*	*uo*	*a+b*	*uo*	*uo*	*uo*	*a+b*
+SDS 10 mM	α-helix	57	77	61	67	27	97	27	55
	β-sheet	5	8	2	2	24	3	24	4
	Turn	26	0	19	1	23	0	23	16
	Unordered	12	15	18	30	26	0	26	25
	3º Structure	*a+b*	*a+b*	*a+b*	*a+b*	*a+b*	*a+b*	*a+b*	*a+b*

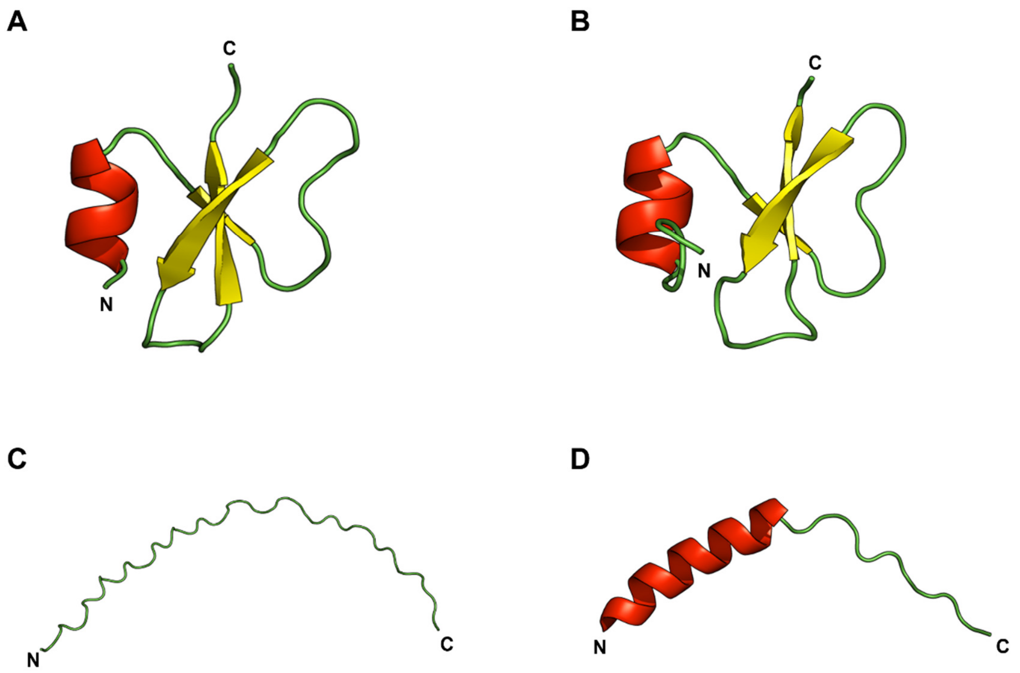

Supplementary Figure S1. Three-dimensional structure of BNBD1 **(a)**, BNBD3 **(b)**, Bac5 segment 131-173 **(c)** and BMAP27 segment 132-158 **(d)** according to their PDB. Tertiary structures are represented using PyMOL software where α-helix is red, β-strand is in yellow, and loop is in green.

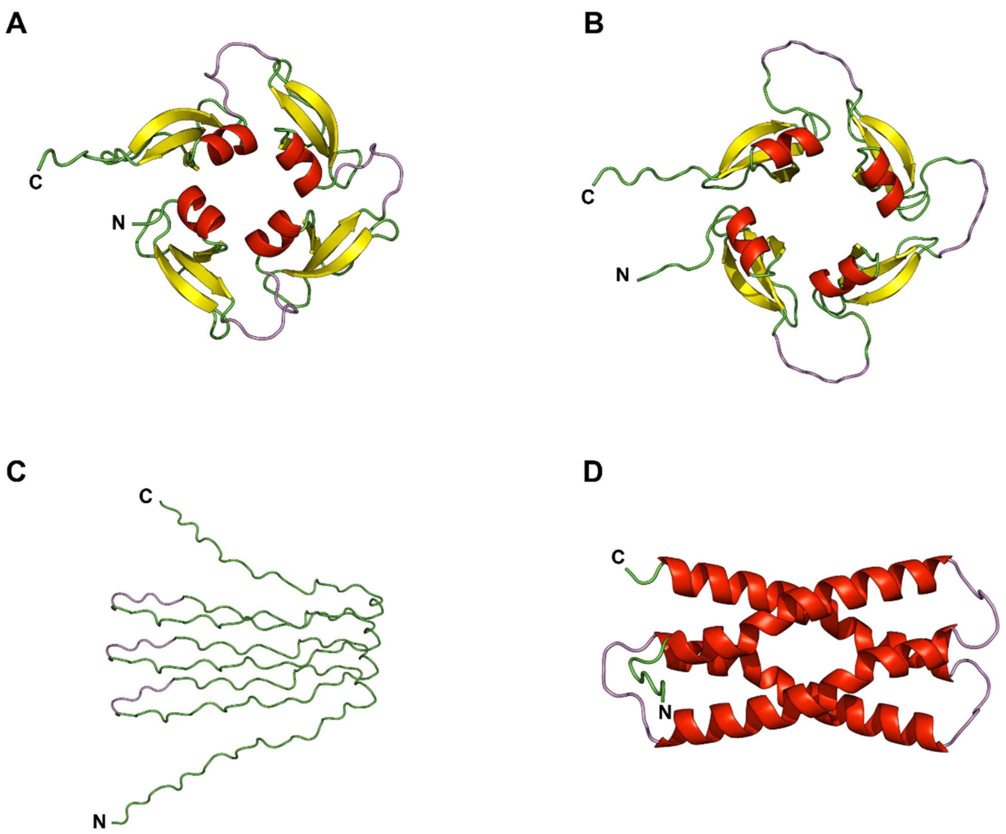

Supplementary Figure S2. Three-dimensional structure of rBNBD1x4 **(a)**, rBNBD3x4 **(b)**, rBac5x4 segment **(c)** and rBMAP27x4 **(d)** as predicted by AlphaFold 3 [94]. Tertiary structures are represented using PyMOL software. α-helix in red, β-strands in yellow, loops in green, and the linker (GGSSRSS) in violet.

References

94 Abramson, J.; Adler, J.; Dunger, J.; Evans, R.; Green, T.; Pritzel, A.; Ronneberger, O.; Willmore, L.; Ballard, A.J.; Bambrick, J.; et al. Accurate Structure Prediction of Biomolecular Interactions with AlphaFold 3. *Nature* **2024**, *630*, 493–500. https://doi.org/10.1038/s41586-024-07487-w.
